# Action of Alcohol

**Published:** 1865

**Authors:** 


Action of Alcohol.—The *Chem. News* says, M. Perrin has experimented on the influence of alcoholic drinks taken in moderate quantities on nutrition. He found that less carbonic acid was exhaled from the lungs when wine was taken. His estimations of urea showed nothing particular. He believes with Dr. Smith and others, that alcohol is not assimilated, but it affects nutrition by lessening the expenditure of material.

